# Internal radiation dose estimates in organs of Wistar rats exposed to sprayed neutron-activated ^31^SiO_2_ microparticles: first results of international multicenter study

**DOI:** 10.1093/jrr/rrae063

**Published:** 2024-10-10

**Authors:** Valeriy Stepanenko, Hitoshi Sato, Andrey Kaprin, Nariaki Fujimoto, Almagul Kushugulova, Sergey Ivanov, Peter Shegay, Viktoria Bogacheva, Alexey Petukhov, Kassym Zhumadilov, Evgenia Ostroumova, Hiroshi Yasuda, Noriyuki Kawano, Megu Ohtaki, Satoru Endo, Aya Sakaguchi, Laura Chulenbayeva, Nurislam Mukhanbetzhanov, Masaharu Hoshi

**Affiliations:** A. Tsyb Medical Radiological Research Center, Branch of the National Medical Research Radiological Centre of the Ministry of Health of the Russian Federation, 4 Koroleva St., Obninsk, Kaluga Region 249036, Russian Federation; Ibaraki Prefectural University of Health Sciences, 4669-2 Ami, Ami-machi, Inashiki-gun, Ibaraki 300-0394, Japan; National Medical Research Radiological Centre of the Ministry of Health of the Russian Federation, 4, Koroleva Str., Obninsk, Kaluga Region 249036, Russian Federation; Peoples’ Friendship University of Russia, 6 Miklukho-Maklaya St., Moscow 117198, Russian Federation; P.A. Hertzen Moscow Oncology Research Institute-Branch of the National Medical Research Radiological Centre of the Ministry of Health of the Russian Federation, 2nd Botkinsky Drive 3, Moscow 125284, Russian Federation; Research Institute for Radiation Biology and Medicine, Hiroshima University, 1-2-3, Kasumi, Minami-ku, Hiroshima 734-8551, Japan; Nazarbayev University, 53 Kabanbay Batyr Avenue, Astana City 010000, Republic of Kazakhstan; A. Tsyb Medical Radiological Research Center, Branch of the National Medical Research Radiological Centre of the Ministry of Health of the Russian Federation, 4 Koroleva St., Obninsk, Kaluga Region 249036, Russian Federation; Peoples’ Friendship University of Russia, 6 Miklukho-Maklaya St., Moscow 117198, Russian Federation; National Medical Research Radiological Centre of the Ministry of Health of the Russian Federation, 4, Koroleva Str., Obninsk, Kaluga Region 249036, Russian Federation; A. Tsyb Medical Radiological Research Center, Branch of the National Medical Research Radiological Centre of the Ministry of Health of the Russian Federation, 4 Koroleva St., Obninsk, Kaluga Region 249036, Russian Federation; A. Tsyb Medical Radiological Research Center, Branch of the National Medical Research Radiological Centre of the Ministry of Health of the Russian Federation, 4 Koroleva St., Obninsk, Kaluga Region 249036, Russian Federation; International Department of Nuclear Physics, New Materials and Technology, L. N. Gumilyov Eurasian National University, 13 Munaitpasova St., Astana City 010008, Republic of Kazakhstan; Environment and Lifestyle Epidemiology Branch, International Agency for Research on Cancer/WHO, 25 Avenue Tony Garnier, Lyon 69366, France; Research Institute for Radiation Biology and Medicine, Hiroshima University, 1-2-3, Kasumi, Minami-ku, Hiroshima 734-8551, Japan; The Center for Peace, Hiroshima University, Higashisenda-machi 1-1-89, Naka-ku, Hiroshima 730-0053, Japan; The Center for Peace, Hiroshima University, Higashisenda-machi 1-1-89, Naka-ku, Hiroshima 730-0053, Japan; Graduate School of Advanced Science and Engineering, Hiroshima University, 1-4-1, Kagamiyama, Higashi, Hiroshima 739-8527, Japan; Institute of Pure and Applied Sciences, University of Tsukuba, 1-1-1 Tennodai, Tsukuba, Ibaraki 305-8577, Japan; Nazarbayev University, 53 Kabanbay Batyr Avenue, Astana City 010000, Republic of Kazakhstan; Nazarbayev University, 53 Kabanbay Batyr Avenue, Astana City 010000, Republic of Kazakhstan; The Center for Peace, Hiroshima University, Higashisenda-machi 1-1-89, Naka-ku, Hiroshima 730-0053, Japan

**Keywords:** dosimetry, internal irradiation, Wistar rats, neutron activation, ^31^SiO_2_, radioactive microparticles, atomic bombing

## Abstract

Neutron-activated ^31^Si is an almost pure beta emitter and is one of the short-lived radionuclides, including beta-gamma emitter ^56^Mn, which were created in a form of residual radioactivity in the early period after the atomic bombing of Hiroshima and Nagasaki. The features of the biological effects of internal irradiation by these radionuclides are a subject of scientific discussions and research. The publication presents data on internal radiation doses in experimental Wistar rats that were exposed to sprayed neutron-activated microparticles of ^31^SiO_2_. Doses of internal radiation could be conditionally divided into three groups according to their values. It has been found that elevated values of internal radiation doses in rats’ organs/tissues as a result of exposure to sprayed ^31^SiO_2_ microparticles with initial activity of 3.2 × 10^7^ Bq varied from 10 to 120 mGy (eyes, lungs, skin, stomach, jejunum, large intestine). The moderate dose values were in the range from 1.9 to 3.7 mGy (trachea, esophagus, ileum). The smallest doses were received by the kidney, testis, blood, cerebellum, heart, liver, cerebrum, bladder, spleen and thymus (from 0.11 to 0.94 mGy). The obtained data are important for interpreting the results of ongoing and planned biological experiments with ^31^SiO_2_ microparticles—in comparison with the previously published data on features of biological effects caused by beta-gamma emitting ^56^MnO_2_ neutron-activated microparticles.

## INTRODUCTION

There are a number of short-lived neutron-activated radionuclides, which are significant part of residual radioactivity as dose-forming factors in the early stage after the atomic bombing of Hiroshima and Nagasaki on 6 and 9 August 1945 [1–4]. Silicon-31 (^31^Si) is one of these radionuclides. Scientific research and discussions on the impact of exposure in relation to biological effects from the residual radioactivity following the A-bombing are ongoing [5–7]. The reason for that is an earlier publication on harmful radiation effects observed in the persons who came to Hiroshima shortly after the nuclear detonation, but who were not exposed to the initial gamma-neutron radiation at the moment of explosion [8–12]. As a result, there is a reason to believe that exposure of these individuals to the residual radioactivity in the early stage after the atomic bombing was the main cause of the reported health effects. Meanwhile, in very early and even in more recent documents, little attention was given to the residual radiation after the atomic bombardments of Hiroshima and Nagasaki [13–16].

That is why, international studies on features of biological effects and assessments of internal radiation doses in experimental rats and mice exposed to neutron-activated radiation from sprayed microparticles with short-lived radionuclides were carried out now [7, 17–29] and ongoing. It should be pointed out that radiobiological studies on laboratory animals are not intended directly to exact model all conditions of radioactive particles fallout from the atomic-bomb explosions, which is impossible in experimental situation. On the other hand, short-lived neutron-activated radionuclides ^31^Si (T_1/2_ = 2.62 h) and ^56^Mn (T_1/2_ = 2.58 h) are belonging to the main dose-forming factors from neutron-activated residual radioactivity during the first hours after atmospheric atomic explosions [1, 2].

It was found in radiobiological experiments with internal irradiation to ^56^Mn0_2_ microparticles, that rat’s lung tissue was severely damaged by exposure to ^56^Mn [17, 18], despite a rather low radiation dose (the averaged over volume of rats lungs absorbed dose of internal radiation is less than 0.1 Gy [19–23]). Significant biological impact of internal exposure to neutron-activated microparticles with ^56^Mn was found for other radiobiological effects in experiments with rats and mice of different strains [17, 24–28].

The radiation from ^31^Si consists of intensive short-range beta particles (100%). Gamma-radiation of ^31^Si has very low intensity (0,07%). This is principal difference from the radiation of ^56^Mn, which includes intensive beta particles (100%) and intensive gamma rays (143%). These differences can lead to differences in local absorbed dose around ^31^SiO_2_ and ^56^MnO_2_ microparticles entered to biological microstructures. As it was shown in [18, 29], local radiation dose in biological microstructures irradiated by ^56^MnO_2_ microparticles plays significant role in observed biological effects.

Here, we report the data on internal radiation doses averaged over volumes of various organs and tissues of experimental rats exposed to sprayed neutron-activated ^31^SiO_2_ microparticles—with almost pure beta emitting ^31^Si. This is the first stage of internal dose estimates related to ^31^Si. As to the peculiarities of local dose distributions in biological microstructures irradiated by ^31^SiO_2_ particles, the corresponding investigations are ongoing.

Results of internal dose estimates related to ^31^SiO_2_ particles exposure are important for comparative analysis of biological effects caused by internal irradiation from the radioactive microparticles, which have different types of radiation: almost pure beta particles radiation from ^31^Si vs mixed beta-gamma radiation from ^56^Mn [7, 30].

## MATERIALS AND METHODS

### Irradiation and experimental animals

High-purity (>99.9%) silicon dioxide microparticles (SiO_2_) (Admatechs, Japan) with an average diameter of 2.4 μm were used as a substance for neutron activation. Neutron irradiation of SiO_2_ microparticles was carried out at the Research Nuclear Reactor WWR-K of the Institute of Nuclear Physics (Almay, Kazakhstan [31]) by thermal neutrons flux of 7.26 × 10^13^ neutron/(cm^2^ × s) with duration of irradiation equal to 300 s. The activity of ^31^Si (T_1/2_ = 157.3 minutes) obtained as a result of neutron activation was equal to 4.92 × 10^7^ Bq per 1 g of SiO_2_ microparticles.

Animal experiment was performed in accordance with the Approval by Ethics Committee of Kazakh National Medical University named after S.D. Asfendiyarov (Protocol # 7 (1457), 7 September 2023, Almaty, Republic of Kazakhstan), and in accordance with the Directive 2010/63/EU of the European Parliament and the Council of 22 September 2010 on the protection of animals used for scientific purposes [32].

**Fig. 1:**
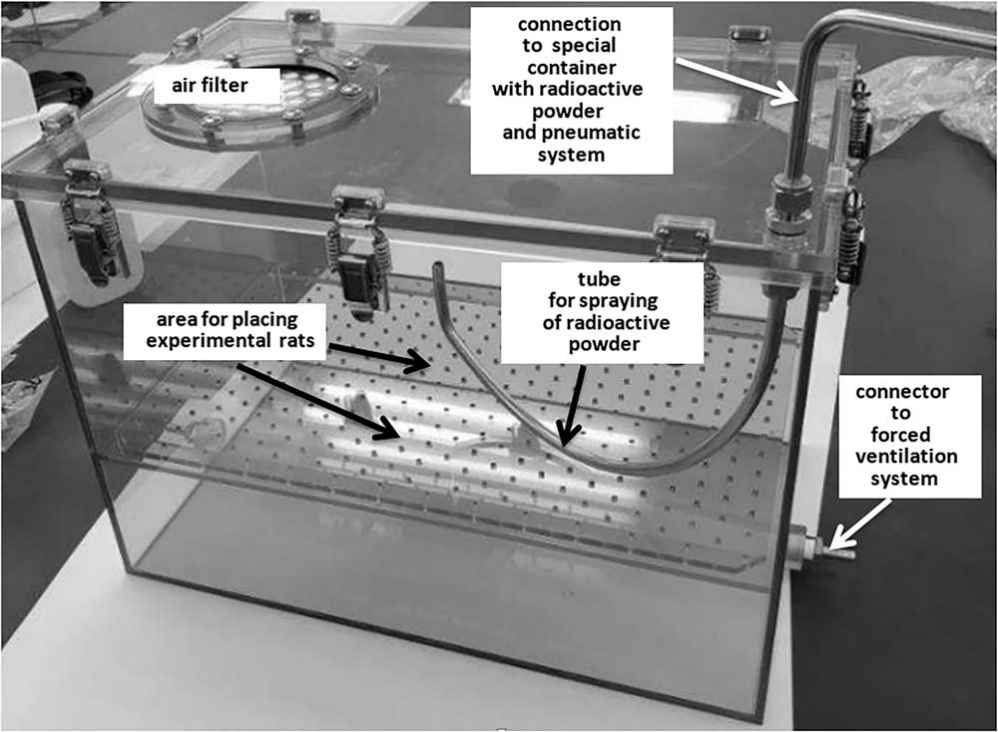
General view of the box used for spraying of neutron-activated ^31^SiO_2_ powder over the experimental animals.

Neutron-activated ^31^SiO_2_ powder was sprayed over the experimental animals using a special exposure box with forced ventilation (Fig. 1) similar to the box that was used in experiments with neutron-activated ^56^MnO_2_ microparticles [17, 21–23]. The features of this box were the following: (i) the main part of the construction was an isolated box where experimental animals were placed, preventing a release of radioactive microparticles into the environment; (ii) the box was connected with a forced ventilation system and equipped with an air filter that were necessary to ensure the breathing of animals; (iii) the box was connected with a pneumatic system, which, with a stream of air, sprayed radioactive powder over animals in the box. The sizes of the exposure box were enough to place inside up to seven experimental rats having a length of 85 cm, width of 55 cm, and height of 60 cm. For the exposure to sprayed radioactive powder (^31^SiO_2_ microparticles), two identical boxes were used. Thirteen experimental rats were exposed simultaneously: seven rats were placed in the first box for subsequent biological studies and six rats were placed in the second box, for both biological and dosimetric studies. Outbreed Wistar male rats, 10 weeks old, and with mean body mass of 311 g ± 7.6 g (SD), were used for the experiment. These rats were bred in the animal research facility of the Kazakh National Medical University named after S.D. Asfendiyarov and their health status corresponded to the specific pathogen-free (‘SPF’) conditions [33]. Exposure to SiO_2_ microparticles began 95 minutes after the completion of neutron activation. In each box with the experimental rats, 1000 mg of ^31^SiO_2_ powder was sprayed. The activity of sprayed ^31^SiO_2_ microparticles at the start of exposure was equal to 3.2 × 10^7^ Bq in each box. The duration of rats’ keeping in the box used for spraying of neutron-activated ^31^SiO_2_ was equal to 90 minutes. After 90 minutes, experimental animals were removed from the box.

Ten minutes after the end of exposure, all rats were euthanized by whole blood removal from an abdominal artery under anesthesia induced by isoflurane [34]. After euthanasia, the rats were dissected to obtain organ/tissue samples. Each extracted sample was minced with a scalpel so that thin layers of biological samples (1.9 mm thickness) could be obtained for the subsequent radiometry of ^31^Si beta radiation. Surgical extraction and preparation of biological samples were performed during 16–123 minutes after rats’ removal from the boxes used for exposure. To avoid cross-contamination of tissue and organ samples, new tweezers, scissors and scalpels, which did not have radioactive contamination, were used for each procedure. Each crushed biological sample was placed in a thin-walled labeled plastic dish with a known weight. The gross weight of each plastic dish with the sample was measured on high-precision analytical balances, and then the net weight of the biological sample was recorded. After this, each weighted dish with a sample was promptly transferred for radiometry of ^31^Si beta radiation. Radiometry of biological samples was performed 115–325 minutes after the beginning of exposure. The time periods of all procedures indicated above were recorded to take into account the decay of short-lived ^31^Si. Radiometry was carried out in a separate room. Walls of the room for radiometry were shielded with lead sheets of 5 mm thickness and 1.5 m height.

### Methodology of dose estimates

**Fig. 2:**
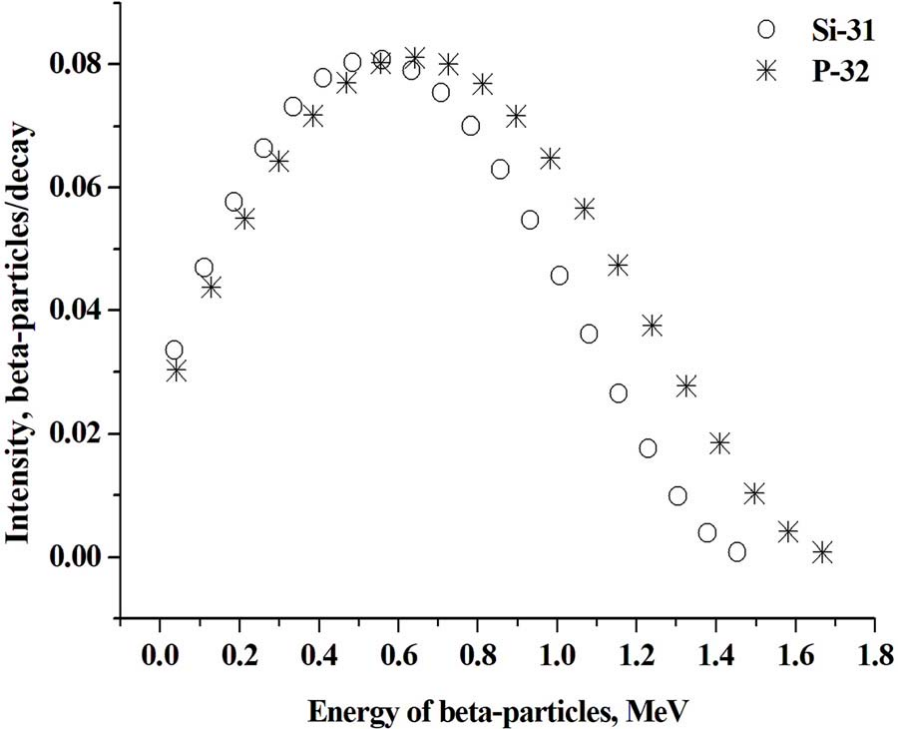
Comparison of ^31^Si and ^32^P beta emission spectra [40–42] approximated by 20 energy lines. The maximum and average energies of ^31^Si beta particles are of 1.492 and 0.595 MeV, respectively. The maximum and average energies of ^32^P beta particles are of 1.709 and 0.695 MeV, respectively.

Estimates of absorbed doses of internal radiation were performed in accordance with the methodology of the Medical Internal Radiation Dose Committee (MIRD) [35] using the results of measurements of ^31^Si-specific activity in the rats’ organs and tissues, as well on the basis of calculated absorbed fractions (AF) of energy emitted by beta particles from ^31^Si accumulated in organs and tissues. At AF calculations, the ^31^Si beta emission energy spectrum was approximated by 20 electron energy lines using the Radiation Dose Assessment Resource (RADAR) data (Fig. 2). AFs were calculated using Monte Carlo (MC) program (MCNP-4C) [36], and an age-dependent mathematical phantom of an experimental rat was used as well [37, 38]. It was assumed that the chemical composition and density of rat’s organs and tissues are similar to those of the human body [39].

## RESULTS

### ^31^Si beta particles counting with developed stand and calibration of counter

Special stand equipped with high-sensitive ‘Ranger Exp’ Geiger-Mueller (GM) counter (S.E. International, Inc.) was developed to measure beta radiation from biological samples.

**Fig. 3:**
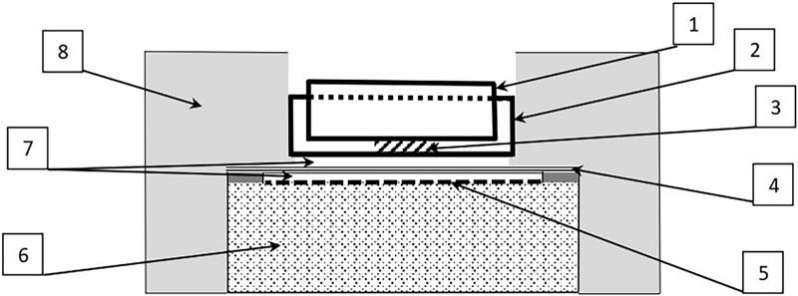
Schematic view of the stand to measure beta radiation from a biological sample by GM counter. There are upper dish (1) and lower dish (2), respectively, which were located in the stand to measure ^31^Si beta radiation from a biological sample. A crushed biological sample (3) of 1.9 mm thickness was placed between the bottom of the upper dish and bottom of the lower dish. Dishes were made of polymethyl methacrylate (PMMA) of 0.74 mm thickness and 1.19 g/cm^3^ density. Polyethylene protective sheet (4) of 0.1 mm thickness and 0.93 g/cm^3^ density, was designed to protect the thin mica window (5) of GM probe (6). Mica window had 1.4–2.0 mg/cm^2^ areal density and 45 mm effective diameter. There were air gaps (7) with a thickness of 2.5 mm (upper) and 1.75 mm (lower), respectively. The holder (8) of the entire construction was made of PMMA.

Figure 3 shows a scheme describing all parts of the measurement stand used.

The Geiger-Mueller counter with an external Halogen-quenched, uncompensated GM probe, was calibrated as follows.

At the first stage, ^32^P source was used for calibration. The choice of ^32^P was made because this radionuclide, like ^31^Si, is a pure beta emitter, and the shapes of beta particle energy spectra for both of these radionuclides are similar as shown in Fig. 2 (with some differences in the mean and maximum energies of beta particles) [41]. Radionuclide ^32^P is more convenient for calibration measurements, because its half-life (T_1/2_ = 14.26 days) significantly exceeds the half-life of the short-lived ^31^Si (T_1/2_ = 157.3 minutes).

To prepare ^32^P source for GM counter calibration, a standard solution with ^32^P (PerkinElmer, Inc.) was used. Different standard activities of ^32^P (ranged from 30 to 330 Bq) in a form of solution were dropped, dried, and adhered on the surface of polymethyl methacrylate (PMMA) plates. The obtained standard ^32^P sources in a form of very thin layers with diameter of 5 mm adhered to the surface of this PMMA plate were placed in the same stand, which was used for measurements of ^31^Si activity in biological samples (Fig. 3). Measurements of counts from standard ^32^P sources were carried out by ‘Ranger Exp’ GM counter. The geometry of ^32^P standard sources measurements was similar to the geometry of radiometry of ^31^Si beta radiation from biological samples, but with only one exception: unlike biological samples with mean thickness of 1.9 ± 0.03 mm (SD), the ^32^P standard sources were in a form of layers with very small thickness, which were adhered to the surface of PMMA plate. It was assumed that the absorption of ^32^P beta particles in these very thin layers of the standard sources was negligible.

At the second stage, the value of the calibration coefficient, obtained as a result of the measurements of beta particle counts from thin standard ^32^P sources, was recalculated for 1.9-mm thick biological samples with beta-emitting ^31^Si. This recalculation was performed using Monte-Carlo method [36]. When recalculating by MC method, we took into account the self-absorption of ^31^Si beta particles in a 1.9-mm thick with a diameter of 5 mm biological sample, backscattering of ^31^Si beta particles from a PMMA plate, as well as differences in beta particles energy spectrum of ^32^P and ^31^Si (Fig. 2). That is, in this recalculation, a biological sample of 1.9 mm thickness was considered as a source of ^31^Si beta particles. It was assumed that ^31^SiO_2_ microparticles are homogeneously distributed within the volume of biological sample. As a result of MC recalculation, the value of calibration coefficient for 1.9 mm ± 0.03 mm-thick biological sample considered as a ^31^Si radioactive source was estimated equal to 0.12 ± 0.007 (SD) counts/s per 1 Bq of ^31^Si.

### Results of dose estimations

According to the MIRD’s methodology, internal radiation doses were calculated taking into account accumulated activity of ^31^Si in all considered organs and tissues of rats. The measured specific activity of ^31^Si in rats’ organs and tissues was recalculated from the time of activity measurement to the moment of the beginning of animals’ irradiation, taking into account the physical half-life of ^31^Si. The accumulated internal radiation doses were estimated for the time period from the start of exposure to infinity (until the complete decay of ^31^Si).

It must be emphasized that radioactive microparticles of ^31^SiO_2_ were sprayed over experimental animals using a pulse of compressed air from a special pneumatic system (Fig. 1). The duration of this pulse was 20 seconds. Therefore, it was assumed that the inhalation intake of microparticles into the animals’ respiratory system was short-term (not more than 1 minute)—as long as the ^31^SiO_2_ microparticles were suspended in the air of the box.

After spraying, radioactive powder was deposited on the walls and floor of the box, as well as retained by the hair of the animals’ skin. Radioactive microparticles deposited on walls and floors and retained on the hair were swallowed by experimental rats in the process of cleaning and grooming. The duration of high activity of rats on cleaning and grooming was observed for about 20 minutes after spraying the radioactive powder. The animals then calmed down and their behavior became less active, and the cleaning process ended. Therefore, it was assumed that the intake of radioactive particles by ingestion (swallowing) was also relatively short-term (about 20 minutes), in comparison with decay of ^31^Si (T_1/2_ = 157.3 minutes).

As a result, dose calculations were performed assuming a single intake of the activity. This assumption is consistent with duration of intake by inhalation (estimated duration of inhalation is about 1 minute). Regarding duration of intake by swallowing, assumption about single intake is less consistent—as far as duration of swallowing was estimated equal to 20 minutes compared to the half-life of ^31^Si equal to 157.3 minutes. Therefore, the assumption of a single intake of radioactive particles through ingestion should be considered somewhat conservative. Nevertheless, the assumption about single intake by swallowing was considered acceptable, since the duration of ingestion of about 20 minutes is ~eight times less than the half-life of ^31^Si.

It was suggested as well, similar to [21–23], that the physical decay of ^31^Si is essentially faster in comparison with further biological redistribution of ^31^SiO_2_ microparticles in the experimental rat’s organism.

**Fig. 4:**
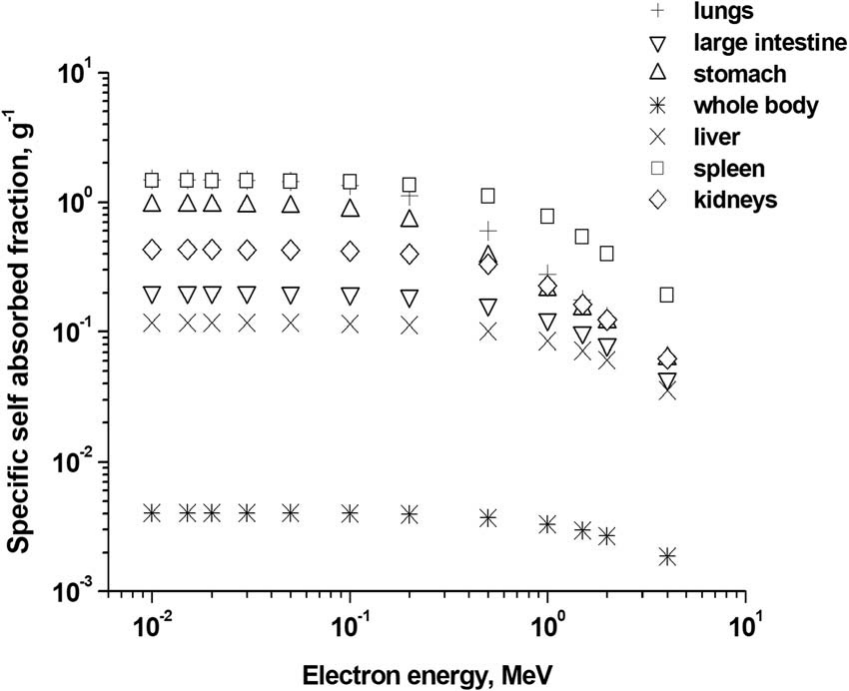
Example of ‘Specific self-absorbed fraction’ values in various organs of experimental rat vs energy of electrons, MeV. The term ‘Specific self-absorbed fraction’, g^−1^, means AF per ‘target’ organ’s mass for a case when ‘source’ organ and ‘target’ organ are the same.

**Fig. 5:**
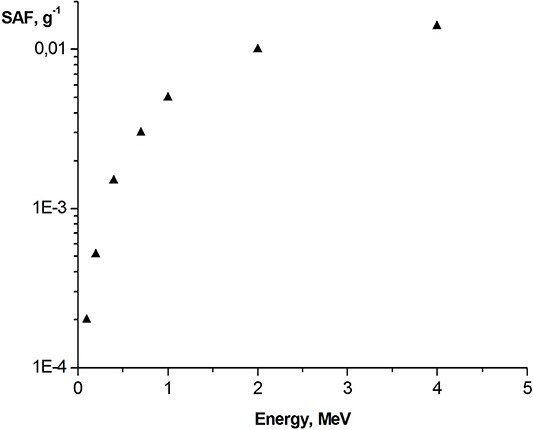
Example of specific absorbed fractions (SAF) values, g^−1^, vs energy of electrons, MeV: rat’s liver (‘source’) irradiating stomach (‘target’).

It was assumed, that ^31^SiO_2_ microparticles were homogeneously distributed in organs and tissues. It means that the accumulated doses of internal radiation were the average doses over the volumes of organs/tissues. Figures 4 and 5 show typical values of specific AFs of energy in the volumes of various organs of a rat when they were irradiated to electrons with different energies.

Table 1 shows the results of absorbed dose estimations in considered organs and tissues of experimental rats.

**Table 1:** ^31^Si activity concentrations (A_0_) and corresponding absorbed doses (D) in organs and tissues of Wistar rats

Organs	A_0_, kBq/g (±2 SD)	D, mGy (±2 SD)
Blood	0.07 ± 0.02	0.19 ± 0.06
Thymus	0.32 ± 0.08	0.94 ± 0.3
Trachea	0.62 ± 0.2	1.9 ± 0.6
Lungs	3.5 ± 0.9	11 ± 4
Heart	0.071 ± 0.02	0.23 ± 0.08
Liver	0.067 ± 0,02	0.28 ± 0.1
Spleen	0.12 ± 0.04	0.46 ± 0.2
Kidney	0.035 ± 0.01	0.11 ± 0.04
Esophagus	0.81 ± 0.3	2.6 ± 0.9
Stomach	18 ± 6	24 ± 8
Jejunum	27 ± 7	36 ± 12
Ileum	1.1 ± 0.3	3.7 ± 2
Large intestine	67 ± 20	120 ± 40
Bladder	0.15 ± 0.04	0.34 ± 0.1
Testis	0.059 ± 0.02	0.14 ± 0.05
Skin	9.9 ± 3	19 ± 6
Thigh muscle	0.15 ± 0.04	0.36 ± 0.1
Eyes	3.1 ± 0.8	10 ± 3
Cerebrum	0.14 ± 0.04	0.31 ± 0.1
Cerebellum	0.091 ± 0.03	0.21 ± 0.07

## CONCLUSIONS AND DISCUSSION

We estimated the values of internal radiation doses of various organs and tissues of experimental Wistar rats exposed to sprayed neutron-activated ^31^SiO_2_ microparticles. Doses of internal radiation could be conditionally divided into three groups according to their values. It was found that elevated dose values of internal irradiation of rats’ organs/tissues as a result of exposure to sprayed ^31^SiO_2_ microparticles with initial activity of 3.2 × 10^7^ Bq ranged from 10 to 120 mGy (eyes, lungs, skin, stomach, jejunum, large intestine). Moderate dose values ranged from 1.9 to 3.7 mGy (trachea, esophagus, ileum). The smallest doses were received by the kidney, testis, blood, cerebellum, heart, liver, cerebrum, bladder, spleen and thymus (from 0.11 to 0.94 mGy).

High and moderate levels of radiation doses to the gastrointestinal tract, esophagus, skin, eyes, lungs and trachea could be explained by the following: (i) swallowing of the radioactive particles by the rats due to the natural process of hair’s cleaning and grooming; (ii) a retention of sprayed microparticles on the hair and skin of the rats and (iii) inhalation of radioactive microparticles.

It should be noted that the activity of sprayed neutron-activated ^31^SiO_2_ microparticles used in the study (3.2 × 10^7^ Bq) was about nine times less than the activity of sprayed neutron-activated ^56^MnO_2_ microparticles used in experiments [21, 23]. Moreover, the mean energy of ^31^Si beta particles (0.595 MeV) was less than the mean energy of ^56^Mn beta particles (0,829 MeV). This is the reason why dose estimates related to internal irradiation of rats by sprayed neutron-activated ^31^SiO_2_ is significantly less in comparison with dose estimates in experiments with ^56^MnO_2_ microparticles [21, 23].

It is necessary to emphasize that the peculiarities of internal exposure to radioactive microparticles and the associated radiobiological effects of radiation exposure on animals are the focus of current studies [43–47].

The data obtained in our study are necessary for the interpretation of the results of ongoing and planned biological experiments with various activities of sprayed ^31^SiO_2_ microparticles—for comparative analysis regarding the features of biological effects caused by internal exposure to radioactive microparticles, having different types of radiation.

It should be noted that the estimated values of internal radiation dose are the average doses over volumes of considered organs/tissues of experimental animals. The investigations of features of dose distribution on the level of biological microstructures irradiated by ^31^Si are ongoing now (similar to that, what was done for ^56^Mn [18, 29]).

## Data Availability

All authors are ready to provide data upon request.
